# Drivers and barriers to rural and urban healthcare placement in Ghana: a Delphi study

**DOI:** 10.3389/fpubh.2025.1436098

**Published:** 2025-05-29

**Authors:** Bernard Okoe Boye, Subhash Pokhrel, Kei Long Cheung, Nana Anokye

**Affiliations:** Department of Health Sciences, Brunel University London, Uxbridge, United Kingdom

**Keywords:** e-Delphi, health policy, Ghana, drivers and barriers, consensus

## Abstract

**Objective:**

This study explored the level of consensus on the drivers and barriers influencing doctors’ decisions to work in rural versus urban areas. The study provides insights into systemic issues affecting healthcare workforce distribution in Ghana. Access to medical care is particularly important given the changing demographics of Ghana, including the growth of the older and chronically ill population and the high proportion of older adults living in rural areas.

**Methods:**

A three-round e-Delphi study was conducted among doctors and regional directors of the Ghana Health Service using a seven-point Likert scale. A median score of ≥6 and an interquartile range of ≤1 was used as cutoffs. In total, 47 experts participated in the study. Although 55 initially registered interest, only 47 took part in the first round. By the second and third rounds, 42 experts remained engaged in the study.

**Results:**

Experts reached consensus on 40 descriptors (78%), of which 37 (93%) were considered important. Doctors reached consensus on 11 and 7 important drivers and barriers of rural incentive adoption, respectively, while reaching consensus on 8 important drivers of urban incentive factors. Regional directors reached consensus on 4 and 7 important drivers of rural and urban factors, respectively. Four categorical themes emerged from the analysis. These are financial, professional development and career advancement, work-life balance, and community lifestyle factors.

**Conclusion:**

The contrast in drivers and barriers between rural and urban healthcare workers necessitates tailored policy approaches, resource allocation strategies, and workforce planning efforts to ensure equitable access and quality care across diverse settings and among different sub-populations, especially the growing number of aged and chronically ill.

## Introduction

There is a significant divide between healthcare resources available in urban areas compared to rural regions. The inequitable distribution of health personnel in Ghana, with the majority in the cities, is a significant challenge. This creates poor doctor-to-patient ratios in other regions in the country, especially the regions in Northern Ghana. For example, according to the Ghana Health Service (GHS) 2019 Annual Report, the national doctor-to-patient ratio in 2018 was 1:7058 (1). However, there were significant regional disparities, with rural and underserved areas experiencing more severe shortages. The Northern, Upper East, and Upper West Regions, which are predominantly rural, had doctor-to-patient ratios well above the national threshold, indicating a more acute shortage of doctors. Specifically, the Northern Region had a ratio of 1:9385, while the Upper East and Upper West Regions had 1:21,465 and 1:13,606, respectively. In contrast, the Greater Accra and Ashanti Regions, which have a higher concentration of healthcare facilities and professionals, had more favorable ratios of 1:3035 and 1:5953, respectively (1). These differences are particularly important because of Ghana’s changing demographics. The number and percent of older adults (those aged 65 and above) have increased over the past decades from 215,264 in 1960 (3.2% of the total population) to 1,322,538 in 2021 (about 4.3% of the total projected population) (2, 3) By 2030, the older population is projected to be about 1.6 million, further increasing to 14.1 percent of the total population by 2050 (3, 4). Older adults are likely to have chronic conditions (5), and a higher proportion live in rural than urban areas (6). These demographics make it imperative for Ghana to recruit and retain physicians and other healthcare providers in rural areas.

Rural experience or exposure, supportive work environment, adequate living conditions and essential social amenities, well-equipped schools with qualified teachers, reliable road and transportation networks, electricity, and access to clean water, have been widely cited (7–10) as key factors essential to attract, recruit, and retain doctors and other healthcare workers in remote areas.

The World Health Organization (WHO) has outlined several key strategies to develop, recruit, and retain healthcare workers in rural and remote areas. A major focus is on encouraging students from rural backgrounds to pursue healthcare education, as they are more likely to return and serve their communities. Establishing medical and nursing schools closer to these areas further enhances accessibility and helps bridge the healthcare gap. Practical experience in rural settings is essential for preparing students to work in underserved regions. Integrating rural health topics into medical and nursing curricula ensures that students develop the skills needed to navigate the unique challenges of these environments. Clear career development pathways and professional growth opportunities play a vital role in retaining healthcare workers in rural areas. The WHO emphasizes structured career advancement programs that make rural healthcare a viable and attractive long-term option (11). Together, these strategies form the ‘rural pipeline’ approach—designed to build and sustain a strong healthcare workforce in underserved communities.

Rural pipeline programs have had a significant impact in developed economies (12–16). In Northern Europe, for example, about 67% of healthcare professionals trained through these programs continued working in rural and underserved areas even 5 years after their recruitment (12). In Sub-Saharan Africa (SSA), the concept is still relatively new, but some countries are already seeing positive results. A study found that selecting students from rural backgrounds and providing them with training tailored to rural healthcare needs boosted their confidence, job satisfaction, and commitment to staying in rural areas (17). Other research in SSA has also shown that these programs shape healthcare workers’ attitudes, influence their career choices, and increase their willingness to serve in remote communities (18–20). They have strengthened local healthcare systems by ensuring a steady supply of dedicated professionals. However, making these programs sustainable is not always easy. Financial constraints, infrastructure challenges, and management issues can stand in the way of long-term success. Overcoming these barriers will be crucial in ensuring that rural pipeline programs continue to make a difference where they are needed most.

Ghana’s healthcare system faces a major challenge due to shortages and uneven distribution of healthcare workers, particularly in rural areas. To address this, the Ministry of Health envisioned a healthcare workforce that is adequately staffed, well-trained, and equipped with the necessary skills, competencies, and attitudes. The goal is to ensure that health professionals are equitably distributed, highly motivated, and committed to delivering quality care across both the public and private sectors, including rural and underserved areas (21). While research has explored incentives that encourage medical students and other healthcare professionals, such as nurses and midwives, to pursue rural practice, there remains limited understanding of the specific factors that motivate medical doctors to work and remain in rural Ghana.

This study aims to address that gap by examining the key drivers and barriers influencing doctors’ decisions. Examining the facilitators of health policies is essential to ensuring effective policies that are sensitive and purposeful. This is particularly important given the paucity of research on key drivers and barriers of doctors’ work-placement decisions. Given this, the aim of the study is to explore consensus on the most important drivers and barriers and the extent of this consensus among the implementers (regional health directors) and policy target (doctors). One of the standard approaches in exploring consensus in health literature is a Delphi study (22–24).

## Methods

### Study design

A three-round e-Delphi study (25–27) was conducted among doctors and regional health directors in Ghana to identify and prioritize the drivers and barriers influencing doctors’ work-placement decisions. Microsoft Forms was used to conduct the e-Delphi survey.

### Participants and sampling

Ghanaian professionals encompassing doctors and regional directors working with the Ghana Health Service were selected as experts for this study. Doctors were chosen because they play a critical role in healthcare delivery, and their work placement decisions significantly impact the distribution of healthcare services across the country. Regional directors were included because they are responsible for workforce management and decision-making at the regional level, influencing the deployment and retention of healthcare professionals. Doctors were typically graduates of medical schools in Ghana. Regional directors are mostly doctors with a background in public health.

Purposive sampling was used to select participants. This technique maximized the potential for obtaining comprehensive and detailed information since the sample included individuals who could provide rich and in-depth insights into the facilitators and barriers to the workplace placement decisions. The selection criteria were that the experts who were engaged should have been in active service since 2016. This ensured that participants were well abreast of the Ghanaian health ecosystem. The doctors and regional directors were sent an invitational e-mail through a Gatekeeper (Ghana Health Service). In each round, the experts were invited to respond to the questions in an online survey. A total of 47 experts participated in the study, including 31 doctors and 16 regional directors. The 31 doctors account for approximately 0.73% of the total 4,254 registered doctors in Ghana as of 2022 (28). While this represents a small fraction of the national physician workforce, their insights provide valuable perspectives on factors influencing doctors’ work placement decisions. The 16 regional directors account for 100% of all regional directors of the Ghana Health Service. No incentives or formal endorsements were provided; the response rate was achieved through routine professional engagement with the Ghana Health Service.

### Data collection

Participants were informed about the purpose of the research and their right of withdrawing consent from the study. All participants were requested to complete the informed consent form for the study at the start of the online questionnaire. Study approval was given by the ethics committee of the College of Health, Medicine, and Life Sciences of Brunel University London (42304-LR-Sep/2023–47,156-5).

### Delphi first round

The first-round survey comprised two sections. In the initial section, we examined the demographics of the two groups of experts, including their age, gender, and the environment of their respective facilities. This approach has been used by de Vries, Vahl (26) and Wilson, Knight (29). The second part involved open-ended questions (26) assessing: [1] ‘what made you move to the rural area?’; [2] ‘what made you move to the urban area?’; [3] ‘what made you not to opt for the rural area?’ for the doctor questionnaire. For the regional director’s questionnaire, the questions were: [1] ‘what makes doctors in your region choose the urban areas?’; [2] ‘what are the reasons given by doctors who accepted posting to rural areas?” E-mail reminders were sent to non-responders after 2 weeks within the posting of questions. Descriptive analysis through reporting frequencies and percentages was used to analyze the sociodemographic characteristics of participants. Content analysis was also used in round 1 of the Delphi survey. Through the analysis of gathered responses, a comprehensive list of pertinent measures emerged from the identified drivers and barriers. Subsequently, two researchers meticulously reviewed and amalgamated semantically akin measures. Following a thorough discussion involving a third researcher, consensus was reached on the measures to be included in the subsequent round questionnaire (29–31). Results of round 1 were analyzed per stakeholder group to identify group similarities and differences.

### Delphi second round

In the second round, participants were asked to rate the importance of each factor identified in the first round on a seven-point Likert scale, ranging from 1 (fully disagree) to 7 (fully agree). In addition, experts were invited to optionally provide any other drivers or barriers that might have been missed in the first round. Email reminders were sent to non-responders after 2 weeks of non-response. Results of round two were also analyzed per stakeholder group to identify group similarities and differences. Descriptive analysis through reporting frequencies and percentages was used to analyze the sociodemographic characteristics of participants in round two. Median (Mdn) and interquartile range (IQR) were computed. The median score was calculated and a score of ≥6 was considered important (agreement with the factor being important) (30). IQR was used to gain an indication of the degree of consensus between experts on the factors. An IQR value of ≤1 indicated good consensus among the experts. Factors which received the IQR of ≤1 were removed from the third-round questionnaire. This approach has been used by scholars (25, 26, 30). Statistical Package for Social Sciences (SPSS) was used to calculate averages, standard deviations (SDs), percentages, Mdn scores and IQRs. Content analysis was also used to identify any feedback from round 1.

### Delphi third round

The objective of the third round was to establish the final ranking. All participants who completed the second-round questionnaire were sent an invitation to reassess the criteria that did not achieve agreement during the second round. The questionnaire was tailored to each stakeholder group, as the survey provided controlled feedback of the group’s answer shown as Mdn score. This approach ensured that participants were aware of the particular group’s input while keeping the anonymity of the group. The experts evaluated the list of obstacles and enablers using an electronic poll. In this round, they were given their personal round 2 ratings as well as the panel median rating and summarized remarks for each item. Email reminders were sent to those who do not respond within a span of 1 week.

## Results

Fifty-five (55) experts registered their interest in the study. Thirty-nine (39) of them were doctors and sixteen (16) were regional directors. Forty-seven experts (85.5% response rate) took part in the first round, which consisted of 31 doctors and 16 regional directors. Altogether, 51 distinct factors were recognized and employed as inputs for the second and third round surveys. These consisted of 8 barriers to rural incentive adoption, 25 drivers of rural incentive adoption, and 18 drivers of urban incentive adoption. Four main themes emerged from the factors. The themes were: financial incentives, professional development and career advancement, work-life balance, and community and lifestyle factors. A total of 42 experts were included in the second and third rounds. The response rate for the second round was 89.4% (*n* = 42) and the third round was 100% (*n* = 42). The sociodemographic attributes and participation rates for each stakeholder group are shown in Table 1.

**Table 1:** Socio-demographic characteristics of the experts and response rates.

Expert group	Characteristics	1st round (*n* = 47)	2nd round (*n* = 42)	3rd round (*n* = 42)
Doctors	Number invited	39	31	27
	Number participated (% response rate)	31 (79.5)	27 (87.1)	27 (100)
	Age in years (SD)	58.2 (1.99)	45 (6.72)	45 (6.72)
	Gender			
	Male (%)	17 (54.8)	13 (48.1)	13 (48.1)
	Females (%)	14 (45.2)	14 (51.9)	14 (51.9)
	Setting of facility			
	Rural (%)	11 (35.5)	10 (37)	10 (37)
	Urban (%)	20 (64.5)	17 (63)	17 (63)
Regional directors	Number invited	16	16	15
	Number participated (% response rate)	16 (100)	15 (93.75)	15 (100)
	Age in years (SD)	54.5 (3.27)	53.07 (4.91)	53.07 (4.91)
	Gender			
	Male	14 (87.5)	13 (86.7)	13 (86.7)
	Females	2 (12.5)	2 (13.3)	2 (13.3)
	Setting of regions (predominant)			
	Rural	12 (75)	11 (73.3)	11 (73.3)
	Urban	4 (25)	4 (26.7)	4 (26.7)

### Doctors’ perspective on drivers of rural incentive adoption

After the third round, experts reached consensus on 11 important drivers of rural incentive adoption descriptors, shown in Table 2. A consensus was reached on only one important financial incentive theme (higher salaries or bonuses for rural postings). The median score for opportunities to specialize or subspecialize in rural medicine was higher than the other professional development and career advancement theme descriptors indicating higher importance. Moreover, access to continuing medical education opportunities and recognition and awards for rural practice excellence were deemed important as well. Experts reached consensus that supportive and collegial work environment is not an important professional development and career advancement descriptor. Consensus was reached for all the descriptors of the work-life balance theme. Opportunities for community engagement and leadership, as well as a sense of purpose and fulfillment derived from serving underserved communities, were deemed the most important descriptors. For community and lifestyle theme, affordable housing and cost of living was the most important descriptor. Overall, opportunities to specialize or subspecialize in rural medicine, opportunities for community engagement and leadership, a sense of purpose and fulfillment derived from serving underserved communities, and affordable housing and cost of living were the most important descriptors on which consensus were reached.

**Table 2:** Importance and consensus on doctors’ perspective on drivers of rural incentive descriptors.

Themes	Descriptors	R2:Mdn	R2:IQR	R3:Mdn	R3:IQR
Financial incentives	Higher salaries or bonuses for rural postings*	6	1	-	-
	Student loan repayment assistance programs	6	2	6	2
	Relocation allowances or housing subsidies	6	2	6	2
	Tax breaks for rural practice	6	2	6	2
	Allowances to purchase vehicle	6	2	6	2
Professional development and career advancement	Access to continuing medical education opportunities*	6	1	-	-
	Mentorship and support from experienced rural physicians	6	2	6	2
	Opportunities to specialize or subspecialize in rural medicine*	7	1	-	-
	Participation in rural research or clinical trials	6	2	6	2
	Recognition and awards for rural practice excellence*	6	2	6	1
Work-life balance	Flexible work schedules, shorter commute times and reduced call frequency*	6	1	-	-
	Supportive and collegial work environment	5	1	-	-
	Opportunities for community engagement and leadership*	7	1	-	-
	Access to outdoor activities, recreational facilities, and cultural attractions*	6	1	-	-
	A sense of purpose and fulfillment derived from serving underserved communities*	4	3	7	1
Community and lifestyle	Strong sense of community and belonging among rural residents*	6	2	6	1
	Safe and family-friendly living environment*	6	3	6	1
	Affordable housing and cost of living*	7	1	-	-

Mdn, median score; IQR, interquartile range. *, Consensus (IQRs ≤1) on important items (Mdn ≥ 6).

### Doctors’ perspective on barriers of rural incentive adoption

In total, experts reached consensus on seven important barriers of rural incentive adoption descriptors, shown in Table 3. There was no consensus on poor drinking water, although it was considered important. Poor road networks and little opportunity to do extra work at private health facilities for extra income were the most important barriers on which consensus was reached.

**Table 3:** Importance and consensus on doctors’ perspective on barriers of rural incentive descriptors.

Themes	Descriptors	R2:Mdn	R2:IQR	R3:Mdn	R3:IQR
Poor infrastructure	Poor road networks*	6	2	7	1
	Poor drinking water	6	3	6	2
	Unavailable electricity*	6	1	-	-
	Substandard educational facilities for wards*	6	1	-	-
Limited source of extra work	Little opportunity to do extra work at private health facilities for extra income*	7	1	-	-
Career development	Inadequate professional support and guidance as a result of few specialists in rural areas*	6	1	-	-
	Few opportunities to specialize since most rural facilities offer primary care compared to the big hospitals in the cities with specialist departments that offer training*	6	1	-	-
Road map	No assurance of release from rural duty post in the future*	6	1	-	-

Mdn, median score; IQR, interquartile range. *, Consensus (IQRs ≤1) on important items (Mdn ≥ 6).

### Doctors’ perspective on drivers of urban incentive adoption

Overall, experts reached consensus on eight important drivers of urban incentive adoption descriptors, shown in Table 4. The most important of these were access to quality childcare and educational opportunities, availability of safe drinking water, better transport network, and well-resourced health facilities.

**Table 4:** Importance and consensus on doctors’ perspective on drivers of urban incentive descriptors.

Themes	Descriptors	R2:Mdn	R2:IQR	R3:Mdn	R3:IQR
Financial incentives	Higher salaries through ‘locum’*	6	1	-	-
Professional development and career advancement	Opportunity to attend more conferences and workshops*	6	1	-	-
	Mentorship and support from experienced urban physicians*	6	1	-	-
Work-life balance	Access to a variety of cultural and entertainment options	6	2	6	2
	Opportunities for socialization and networking*	6	2	6	1
Better infrastructure	Availability of safe drinking water*	7	1	-	-
	Access to quality childcare and educational opportunities*	7	1	-	-
	Better transport network*	7	1	-	-
	Well-resourced health facilities*	7	1	-	-

Mdn, median score; IQR, interquartile range. *, Consensus (IQRs ≤1) on important items (Mdn ≥ 6).

### Regional directors’ perspective on drivers of rural incentive adoption

Overall, experts reached consensus on four important drivers of rural incentive adoption descriptors, shown in Table 5. The most important of these were the opportunity to complete ‘housemanship’ faster, supportive and collegial work environment, and opportunities for community engagement and leadership. Experts reached consensus that low cost of living is not an important descriptor.

**Table 5:** Importance and consensus on regional directors’ perspective on drivers of rural incentive descriptors.

Themes	Descriptors	R2:Mdn	R2:IQR	R3:Mdn	R3:IQR
Professional development and career advancement	Opportunity to complete ‘housemanship’ faster*	7	1	-	-
Work-life balance	Flexible work schedules, shorter commute times and reduced call frequency*	6	1	-	-
	Supportive and collegial work environment*	7	1	-	-
	Opportunities for community engagement and leadership*	7	1	-	-
Community and lifestyle	Low cost of living	5	1	-	-
	Chance to give back to the rural communities	6	3	6	3
	Enjoy serenity and nature	6	2	6	2

Mdn, median score; IQR, interquartile range. *, Consensus (IQRs ≤1) on important items (Mdn ≥ 6).

### Regional directors’ perspective on drivers of urban incentive adoption

In total, experts reached consensus on seven important drivers of urban incentive adoption descriptors, shown in Table 6. There was no consensus on motivation to learn more from more experienced doctors, although it was considered important. Quality educational facilities for wards were the most important driver on which consensus was reached.

**Table 6:** Importance and consensus on regional directors’ perspective on drivers of urban incentive descriptors.

Themes	Descriptors	R2:Mdn	R2:IQR	R3:Mdn	R3:IQR
Financial incentives	Opportunity to do extra work for more income*	7	1	-	-
Professional development and career advancement	Motivation to learn more from more experienced doctors	5	1	-	-
	Ability to specialize in diverse fields due to complexity of services rendered in urban facilities*	6	1	-	-
Work-life balance	Proximity to family and friends support groups*	7	2	7	1
	No late-night calls except those that are already scheduled*	6	1	-	-
Better infrastructure	Availability of safe drinking water	7	2	7	2
	Better transport network*	7	1	-	-
	Well-resourced health facilities*	7	1	-	-
	Quality educational facilities for wards*	7	0	-	-

Mdn, median score; IQR, interquartile range. *, Consensus (IQRs ≤1) on important items (Mdn ≥ 6).

## Discussion

Although some studies have explored the challenges of health workforce distribution in Ghana, research on the key factors influencing doctors’ decisions to work in specific locations is limited. This study employed an e-Delphi approach to gather expert opinions and establish consensus on the most important drivers and barriers affecting doctors’ work placement choices. The findings provide valuable insights into the factors that shape workforce distribution and retention in the country.

### Drivers of rural incentive adoption

Only doctors mentioned financial incentives as a driver of the rural incentive adoption. However, only one (higher salaries or bonuses for rural postings) was considered important. The primary emphasis on higher salaries or bonuses for rural postings as the pivotal financial incentive in the adoption of rural incentives can be attributed to providing immediate financial benefits and serving as a tangible motivator (32). Healthcare professionals often prioritize incentives that yield more immediate financial gains, especially when facing immediate financial needs or obligations. Regional directors did not consider financial incentives as an important descriptor. Regional directors were, on average, older and occupied more senior administrative roles. This difference in age and career stage may have influenced their perspectives on financial incentives, with regional directors potentially being less financially constrained compared to some of the younger physicians. This contradicts the findings of Kumar, Tian (33) where employers observed that offering monetary incentives was an effective means of luring allied health workers to rural locations. Doctors and regional directors reached consensus on opportunities to specialize or subspecialize in rural medicine as the most important descriptor. The recognition of the significance of this descriptor may stem from the belief that specialized skills and exposure to rural healthcare contributes not only to personal career advancement but also to the overall improvement of healthcare services in rural areas (33).

In the assessment of work-life balance descriptors, both doctors and regional directors reached consensus on supportive and collegial work environment and opportunities for community engagement and leadership as the most crucial. This signifies that they perceive the importance of fostering a positive and collaborative workplace atmosphere, as well as providing opportunities for healthcare professionals to engage with the local community and assume leadership roles within the rural healthcare setting. The importance of a collegial work environment cannot be overstated, as it fosters collaboration, reduces professional isolation, and contributes to job satisfaction. For rural healthcare workers, where professional isolation is a common challenge, supportive workplace relationships can significantly impact their decision to remain in underserved areas (34). Similarly, opportunities for community engagement and leadership roles allow healthcare professionals to establish meaningful connections within their local settings, enhancing their sense of purpose, and commitment. These aspects not only contribute to individual job satisfaction but also promote stronger ties between healthcare workers and the communities they serve, which is critical for the sustainability of rural healthcare systems (35). Flexible work schedules were also emphasized as a key component of work-life balance. The ability to adapt schedules to accommodate personal and professional responsibilities is particularly important in rural areas, where healthcare workers often face unique challenges such as limited childcare options, fewer educational opportunities for their children, and the need to maintain family ties in urban areas. Policies that allow for flexible scheduling can mitigate these challenges and improve retention rates. Research by Ray and Pana-Cryan (36) highlights the role of flexibility in reducing burnout and improving job satisfaction, aligning with the findings of this study. In a systematic review, Keyko, Cummings (37) indicated that nurses who exhibited higher levels of engagement are less inclined to resign or contemplate resigning from their positions in rural locations.

After three rounds of evaluation, doctors involved in the study reached a unanimous consensus on all three descriptors related to community and lifestyle indicators. Notably, the most emphasized aspect was “affordable housing and cost of living.” This indicates a strong collective agreement among doctors regarding the paramount importance of affordable housing and managing the overall cost of living in their decision-making process regarding rural incentive adoption. Conversely, among regional directors, consensus was reached on the descriptor of “low cost of living”; however, it was not deemed as important. This divergence in perception may, in part, reflect differences in the demographic and professional profiles of the two groups. Regional directors, being on average older and more established in their careers and salary schedules, might be less sensitive to financial and lifestyle-related incentives compared to physicians who are earlier in their careers and potentially more affected by immediate financial and living conditions.

### Barriers of rural incentive adoption

In the assessment of barriers to rural incentive adoption, experts identified poor infrastructure as prevalent in rural settings. The most noteworthy barrier unanimously acknowledged was the poor road network. This consensus underscores the pivotal role that well-maintained road play in facilitating healthcare professionals’ accessibility to medical facilities and ultimately influences the attractiveness of rural practice. Similarly, there was consensus among experts on the barriers of inadequate electricity and substandard educational facilities for children. The recognition of inadequate electricity as a significant barrier highlights the impact of power shortages on the delivery of healthcare services. Insufficient access to electricity can compromise patient care and serve as a deterrent for healthcare professionals considering rural placements. The consensus on substandard educational facilities emphasizes the interconnected nature of healthcare workforce decisions with broader community infrastructure, particularly the educational resources available for healthcare professionals’ children. Darkwa, Newman (38) also found that inadequate infrastructure and social amenities hinders the retention of doctors and nurses in rural Bangladesh.

The recognition of limited opportunities for supplementary employment as a barrier to rural incentive adoption highlights a critical challenge faced by healthcare professionals contemplating rural placements: namely, the scarcity of avenues for additional work within private health facilities, which subsequently restricts potential income. This unanimous acknowledgment among experts underscores a collective recognition of the significant influence this barrier exerts on the decision-making processes of healthcare professionals considering rural practice. From an economic standpoint, the constraint imposed by limited opportunities for supplementary income, particularly within private health facilities, directly shapes the financial viability of rural practice. In contexts where such opportunities are scarce, healthcare professionals may encounter financial disincentives, thereby rendering rural placements comparatively less attractive than urban or private sector positions.

The barrier identified as the lack of assurance of release from rural duty post in the future, categorized under the broader theme of an unclear roadmap, points to a significant concern for healthcare professionals considering rural placements. In the Ghanaian medical system, newly qualified doctors are typically required to undertake a mandatory two-year ‘housemanship’ during which they may be posted to rural or underserved areas by the Ministry of Health. The decision regarding the location and duration of these postings is primarily determined by the Ministry of Health, rather than the individual doctor. This system does not always provide formal contractual agreements or clearly defined timelines for reassignment or opportunities for career progression after rural service. As a result, the uncertainty surrounding how long a doctor might remain in a rural post, and under what conditions they might transfer or pursue further training, creates apprehension and affects decision-making regarding rural incentive adoption. Healthcare professionals often seek clarity and assurance regarding their future career trajectories. The lack of a clear roadmap creates uncertainty about the duration of rural assignments, leading to concerns about professional growth, career development, and personal planning. The absence of assurance of release may deter healthcare professionals from committing to rural practice, as it introduces an element of unpredictability that can impact their overall job satisfaction and career decisions. This barrier is not only pertinent to individual healthcare professionals but also has implications for workforce planning and rural healthcare system sustainability. If professionals are unsure about the duration of their rural commitments, it can hinder effective workforce management and lead to challenges in maintaining a stable and skilled healthcare workforce in rural areas.

### Drivers of urban incentive adoption

In the evaluation of drivers influencing the choice of doctors to practice in urban areas, a unanimous consensus was achieved on all descriptors except for access to a variety of cultural and entertainment options. This outcome suggests a shared perspective among doctors on several key factors that significantly impact their inclination toward urban placements. One of the prominently rated descriptors was the importance attributed to access to quality childcare and educational opportunities. Similarly, the availability of safe drinking water indicates the significance doctors place on fundamental infrastructure and services in urban areas. Access to clean and safe drinking water is viewed not only as a necessity but also as a crucial contributor to the overall well-being and quality of life for healthcare professionals and their families.

The emphasis on a well-developed transport network points to the value doctors place on efficient and reliable transportation options in urban areas. Recognizing the demanding nature of medical professions, where timely access to healthcare facilities is imperative, a well-established transport network becomes a key consideration. Furthermore, the high median score for well-resourced health facilities highlights the preference for urban settings equipped with advanced and comprehensive healthcare infrastructure. The availability of cutting-edge and technologically advanced health facilities is considered crucial for doctors in delivering optimal patient care and advancing their professional development. Contrastingly, the lack of consensus on access to a variety of cultural and entertainment options indicates a nuanced understanding among doctors. While these offerings may be considered factors in choosing urban settings, there is not a universal agreement on their primary importance. This variation underscores the diverse priorities and preferences that healthcare professionals may have when opting for urban placements.

Like doctors, regional directors placed importance on a well-developed transport network, highlighting its significance. The emphasis on well-resourced health facilities aligns with doctors’ priorities, indicating a shared recognition among regional directors of the importance of advanced healthcare infrastructure. Additionally, the consensus among regional directors regarding the importance of quality educational facilities for the wards of healthcare professionals mirrors the findings among doctors. This highlights a common understanding among both groups about the significance of education-related amenities for families in the decision-making process for urban placements.

However, the lack of consensus among regional directors on the availability of safe drinking water contrasts with the consensus reached by doctors on this descriptor. This difference may arise from varying perspectives on the perceived importance of water quality as a driver for urban incentive adoption. It could also reflect regional directors’ considerations of other factors that may outweigh concerns related to drinking water, or it may suggest a regional variation in perceived severity. The variation in consensus highlights the complex and nuanced nature of drivers of incentive adoption. Understanding these variations is crucial for tailoring incentive programs to address the unique preferences and priorities of doctors in different regions.

### Situating the drivers and barriers in a global context

Access to timely and appropriate healthcare is widely recognized as a critical factor in improving life expectancy, reducing preventable deaths, preventing diseases, managing chronic conditions, and enhancing overall quality of life. A key contributor to the ongoing challenges in rural health is the persistent lack of access to healthcare services, largely stemming from a shortage of physicians in rural areas (39). Financial incentives play a pivotal role in influencing healthcare professionals’ choice of practice location. A systematic review by Goodfellow, Ulloa (40) identified financial incentives as one of the most critical determinants guiding primary care physicians toward underserved and rural areas. Various policy initiatives have built upon this understanding by offering loan forgiveness, coverage of training costs, or scholarships in exchange for service in these areas. The findings of this study align with such evidence, highlighting a strong consensus on the importance of financial incentives as a key driver of rural incentive adoption. Additionally, this study underscores the role of a sense of purpose and fulfillment derived from serving underserved populations, which was also identified as a consensus factor. This perspective resonates with Hu, Dill (39), who found that physicians practicing in rural areas were significantly motivated by “greater patient needs,” reflecting a mission-driven approach to their career decisions.

Our findings revealed that key factors influencing doctors’ preference for urban areas included access to additional employment opportunities, such as locum work, and the availability of quality schools for their children. These insights suggest that strengthening the rural education system could be a powerful strategy to reduce the disparity in physician workforce distribution between rural and urban regions. This recommendation may also be applicable to attracting other highly skilled professionals to underserved areas. Notably, this finding aligns with Hu, Dill (39), who identified the quality of schools as a significant determinant in physicians’ decisions to practice in urban settings. Moreover, our observation that physicians prioritize career advancement opportunities underscores the critical importance of offering robust professional development programs in rural areas. Such initiatives could help attract, nurture, and retain experienced healthcare providers in underserved regions. This conclusion is further supported by Hu et al. (39), who similarly emphasized the role of career development in shaping physicians’ practice location decisions.

Notably, findings from this study differ from global evidence. Systematic reviews have highlighted rural upbringing, rural exposure, selective enrolment, and rural medical schools as significant factors in attracting and retaining doctors in rural areas (41–43). However, these factors did not emerge as influential in this study, suggesting that the determinants of rural workforce retention in Ghana may differ from those observed in other contexts. These findings provide valuable insights into the unique dynamics shaping workforce distribution and retention in the country.

### Strength and limitation

This study represents the inaugural exploration into the perspectives of experts on crucial drivers and barriers aimed at addressing specific determinants influencing healthcare placement decisions in the country. This research introduces fresh insights into strategies for managing incentive adoption, potentially guiding policy formulation and steering discussions on the trajectory of incentive adoption within the Ghanaian context. Additionally, the utilization of the Delphi approach in this study ensures consensus on a broad array of interventions, drawing from diverse perspectives. This methodological advantage equips policymakers with a range of incentive policy options to address the challenges comprehensively. To bolster the methodological robustness of an investigation, scholars have advocated for a targeted response rate of around 70% for each round (44, 45). In this Delphi study, the response rates across all rounds exceeded the recommended thresholds outlined in the literature. The response rate was 85.5, 89.4 and 100%, respectively, for the first, second and third rounds.

This study, while contributing valuable insights, is not without limitations. The study does not establish causality. The Delphi method, although effective in identifying key factors through expert consensus, is inherently cross-sectional and does not account for temporal changes or causal relationships. As a result, future research incorporating longitudinal designs or advanced statistical techniques, such as randomized control trials or regression analysis, would be necessary to explore the causal relationships between these factors and the recruitment and retainment of doctors.

The study acknowledges the potential influence of cultural, social, and gender dynamics. Cultural factors, such as regional attitudes toward rural healthcare work, community ties, and local practices, could influence the willingness of healthcare workers to accept postings in rural areas. In some regions, traditional norms or community expectations may discourage healthcare workers from relocating, which could impact the uptake of incentive policies. Understanding these regional differences is vital for designing an incentive program that is not only effective but also culturally sensitive.

Furthermore, gender disparities in the healthcare workforce in Ghana warrant further attention. Women in healthcare often face additional challenges related to work-life balance, family responsibilities, and access to professional development opportunities, particularly in rural settings. In regions where gender roles are more traditional, women may face additional barriers when it comes to relocating to rural areas or accepting study leave opportunities that require long-term absences. These gendered barriers can significantly impact women’s decisions to work in remote regions. Future research should investigate the specific cultural, social, and gender-based barriers to an incentive program’s success, as well as their impact on healthcare delivery.

Future research can also explore the perceptions and experiences of healthcare workers regarding workforce policies and interventions such as the Early Study Leave with Pay Incentive Scheme (46). This policy was meant to attract doctors to serve in rural areas. Understanding how this policy was received and experienced in practice could provide deeper insights into drivers and barriers of incentives meant to redistribute doctors to practice in rural Ghana and inform more tailored strategies for improving workforce distribution and retention in Ghana.

### Policy implications

The findings of this study have significant implications for long-term policy planning in Ghana. The results emphasize the need for tailored incentive structures to address regional disparities, as the factors influencing decision making differ between rural and urban areas. A more regionalized approach to incentive structures is recommended, where policies are designed to respond to the unique challenges of healthcare workers in different regions. For instance, regions with fewer healthcare professionals may require larger financial incentives or additional support for healthcare workers to make rural postings more attractive, while urban centers may benefit from policies focused on professional development and career progression. As the older adult population increases, some rural areas may experience even greater percentages of the aged. Regional incentives might be tailored not only to rural versus urban factors but to the proportion of seniors who benefit from healthcare practitioners who enjoy caring for older people and those with chronic illnesses.

Moreover, the study highlights the importance of adapting policies to the evolving needs of the healthcare workforce. Factors such as career advancement and work-life balance play crucial roles in healthcare workers’ decisions to accept or reject the placements. Education can be modified to emphasize work in rural areas or specialization in geriatrics, both of which can alter a doctor’s career choices in favor of practice in rural areas. As the workforce continues to change, policies must be flexible and responsive to shifting expectations. Regular assessments of the workforce’s needs, particularly regarding professional development and the balance between personal and professional lives, will ensure that the incentive scheme remains attractive and relevant. Additionally, the study underscores the importance of incorporating feedback from stakeholders, particularly doctors and regional directors. Given the dynamic nature of healthcare systems, regular consultations with healthcare workers and other relevant stakeholders should be institutionalized. These consultations would help policymakers address emerging challenges and ensure the incentive program remains aligned with the evolving needs of healthcare delivery.

Continuous evaluation of the program’s effectiveness is also crucial. The study recommends establishing a framework for ongoing impact assessments to track both short-term and long-term impact of work-placement decisions. Regular evaluations would provide valuable data to policymakers, enabling them to make informed, data-driven decisions regarding program refinement and resource allocation. Furthermore, financial incentives, particularly the allocation of resources, must be carefully planned to ensure long-term sustainability. Financial incentives have been identified as one of the most significant drivers of rural practice. To ensure continued effectiveness, policymakers should consider how to balance resource allocation across regions while maintaining financial sustainability. Older adults require more time to care for because of their multi-faceted, chronic conditions. Financial rewards for doctors must be adjusted to the characteristics of the population as well as the community.

The findings of this study underscore the importance of targeted strategies to address the maldistribution of healthcare workers, particularly in rural and underserved areas. These insights resonate with the WHO’s Health Workforce Plan, which emphasizes equitable distribution, capacity-building, and sustainability in health workforce development. To align with WHO guidelines, this study recommends periodic workforce assessments to ensure that resource allocation reflects evolving healthcare demands. Furthermore, integrating continuous professional development and career growth opportunities into incentive programs aligns with WHO’s principles of retention and motivation. Additionally, the WHO highlights the need for policies that foster sustainable workforce solutions, including localized training programs, community engagement, and gender-sensitive approaches. Promoting a multi-disciplinary team approach assists doctors in caring for older adults who have complex conditions, with case managers and therapists reducing the burden on the doctor while providing high quality care to seniors. In this context, our findings advocate for tailored policy interventions that address regional disparities while fostering a resilient and adaptive workforce. Embedding these strategies will not only enhance its efficacy but also ensure its sustainability and alignment with global standards for health workforce development.

Finally, this study underscores financial incentives, such as increased salaries and bonuses for rural postings, as critical drivers. These incentives are particularly important in addressing the persistent maldistribution of healthcare workers in Ghana, where rural areas are often underserved due to the reluctance of healthcare professionals to accept postings in these regions. The challenges associated with rural service, including limited professional opportunities, social isolation, and inadequate infrastructure, necessitate financial interventions to attract and retain healthcare workers. To achieve sustainable workforce distribution, it is essential to contextualize these financial incentives within the broader framework of equitable pay. Equitable pay reflects the principle that compensation should correspond to the demands, complexities, and challenges of specific work environments. Rural healthcare workers often face higher workloads, a greater percentage of older patients who require extra time and emotional effort, professional isolation, and significant personal sacrifices, yet urban healthcare workers receive comparable compensation. Addressing such discrepancies is vital for fostering a sense of fairness and ensuring that rural postings are not perceived as punitive or less rewarding. Integrating equitable pay principles into policy frameworks goes beyond the provision of basic financial incentives. For example, targeted salary adjustments that reflect the unique challenges of rural healthcare delivery, coupled with additional supports such as housing allowances, transportation subsidies, and tuition loan forgiveness, can make rural postings more viable and appealing. These measures can also enhance job satisfaction, reduce turnover rates, and create a more sustainable rural healthcare workforce. The alignment of financial incentives with equitable pay principles also has broader implications for healthcare policy. By prioritizing fairness and equity in remuneration, healthcare systems can mitigate workforce disparities and improve service delivery in underserved areas. Furthermore, ensuring that rural healthcare workers feel valued and adequately compensated contributes to improved morale and productivity, which are essential for achieving long-term goals of equitable healthcare access and quality service provision.

## Conclusion

Our study offers a thorough examination of the barriers and drivers influencing doctors’ practice choice in Ghana. Through a nuanced exploration of factors influencing decision-making among healthcare doctors and regional directors, we have unveiled critical elements that mold the dynamics of healthcare workforce distribution and retention. The identified barriers, spanning infrastructure challenges, financial considerations, and work-life balance issues, underscore the diverse hurdles that necessitate attention. Conversely, the drivers, encompassing factors like career development opportunities, community engagement, and well-resourced health facilities, illuminate key catalysts for eliciting positive responses and fostering the practice choice of doctors. In total, experts reached consensus on 40 descriptors (78%) of which 37 (93%) were considered important. Our findings not only enrich the ongoing discourse on healthcare workforce distribution but also offer practical implications for policymakers and healthcare administrators. To enhance the attraction and retention of healthcare professionals in Ghana, strategies must be devised to address the identified barriers while leveraging the most influential motivating factors for healthcare professionals. Furthermore, the study emphasizes the significance of tailoring approaches to consider the varied perspectives of professionals, changing demographics, and innovative policy interventions, thereby ensuring the efficacy, effectiveness and sustainability of workforce policies. Looking ahead, future research efforts should explore specific strategies for designing and implementing workforce interventions, aligning them with the key factors identified in this study. By fostering a deeper understanding of the workforce landscape in Ghana, we aim to support the development of targeted solutions that not only encourage healthcare professionals to serve in rural areas and to care for older adults and others with chronic illnesses but also to promote a motivated and well-distributed health workforce across the country.

## Data Availability

The raw data supporting the conclusions of this article will be made available by the authors, without undue reservation.
